# Therapeutic Exercise Recognition Using a Single UWB Radar with AI-Driven Feature Fusion and ML Techniques in a Real Environment

**DOI:** 10.3390/s24175533

**Published:** 2024-08-27

**Authors:** Shahzad Hussain, Hafeez Ur Rehman Siddiqui, Adil Ali Saleem, Muhammad Amjad Raza, Josep Alemany Iturriaga, Alvaro Velarde-Sotres, Isabel De la Torre Díez

**Affiliations:** 1 Institute of Computer Science, Khwaja Fareed University of Engineering and Information Technology, Abu Dhabi Road, Rahim Yar Khan 64200, Punjab, Pakistan; shahzad.hussain@kfueit.edu.pk (S.H.); adilalisaleem@gmail.com (A.A.S.); ch.amjadraza@gmail.com (M.A.R.); 2 Facultad de Ciencias Sociales y Humanidades, Universidad Europea del Atlántico, Isabel Torres 21, 39011 Santander, Spain; josep.alemany@uneatlantico.es; 3 Departamento de Ciencias de Lenguaje, Educación y Comunicaciones, Universidad Internacional Iberoamericana Arecibo, Arecibo, PR 00613, USA; 4 Universidad de La Romana, Edificio G&G, C/ Héctor René Gil, Esquina C/ Francisco Castillo Marquez, La Romana 22000, Dominican Republic; 5 Facultad de Ciencias de la Salud, Universidad Europea del Atlántico, 39011 Santander, Spain; alvaro.velarde@uneatlantico.es; 6 Departamento de Salud, Universidad Internacional Iberoamericana, Campeche 24560, Mexico; 7 Faculdade de Ciências de Saúde, Universidade Internacional do Cuanza Bairro Kaluanda, Cuito EN 250, Bié, Angola; 8 Department of Signal Theory, Communications and Telematics Engineering, University of Valladolid, 47011 Valladolid, Spain; itordie@gmail.com

**Keywords:** physiotherapy, ultrawide band (UWB) radar, therapeutic exercise, machine learning, opto-electronic sensors, ensemble method

## Abstract

Physiotherapy plays a crucial role in the rehabilitation of damaged or defective organs due to injuries or illnesses, often requiring long-term supervision by a physiotherapist in clinical settings or at home. AI-based support systems have been developed to enhance the precision and effectiveness of physiotherapy, particularly during the COVID-19 pandemic. These systems, which include game-based or tele-rehabilitation monitoring using camera-based optical systems like Vicon and Microsoft Kinect, face challenges such as privacy concerns, occlusion, and sensitivity to environmental light. Non-optical sensor alternatives, such as Inertial Movement Units (IMUs), Wi-Fi, ultrasound sensors, and ultrawide band (UWB) radar, have emerged to address these issues. Although IMUs are portable and cost-effective, they suffer from disadvantages like drift over time, limited range, and susceptibility to magnetic interference. In this study, a single UWB radar was utilized to recognize five therapeutic exercises related to the upper limb, performed by 34 male volunteers in a real environment. A novel feature fusion approach was developed to extract distinguishing features for these exercises. Various machine learning methods were applied, with the EnsembleRRGraBoost ensemble method achieving the highest recognition accuracy of 99.45%. The performance of the EnsembleRRGraBoost model was further validated using five-fold cross-validation, maintaining its high accuracy.

## 1. Introduction

Physiotherapy is the component of modern medical healthcare that provides a mechanism for the development, maintenance, and recovery of human body movement and its functionalities after illnesses or injuries. There are many types of therapeutic exercises that help in curing various illnesses in order to manage pain or prevent diseases. Physiotherapistss and medical experts reccomend therapeutic exercises to subjects with movement impairment and difficulties performing routine tasks due to illness or injury [1]. Stroke, brain injuries, sports injuries, motor disabilities, Parkinson disease, and post-accident injuries need rehabilitation therapeutic exercises. Patients are reccomended therapeutic exercises according to their pace and tolerance levels. The therapeutic training exercises that are adequate for one patient may not be effective for other patients, because they depend on the patient’s condition and on the severity of injuries or illness. There are the major therapeutic exercises categories: range of motion, muscle strengthening exercises, balance exercises, flexibility exercises, and post-surgery exercises [1]. Thus, there is an acute need for physiotherapists and medical experts who can provide rehabilitation facilities to patients affected by certain illnesses or injuries [2]. Physiotherapy rehabilitation is a prolonged process and requires intensive care from a physiotherapist during the treatment session. AI-based systems are being developed to facilitate physiotherapists in the patient as they perform therapeutic exercises, either at therapeutic centers or at home. Several technologies, including optical, inertial, and radiofrequency (RF)-based systems, have been launched in both public and commercial domains for the purpose of tracking and monitoring body motions, postures, and activities. For motion monitoring, camera-based optical systems, like Microsoft Kinect (ToF), are the gold standard. But, they are costly, have privacy concerns, are prone to occlusion, and are preferred for use in lab-based environments. Inertial sensors are comparatively cheap and commercially available, but have calibration and drift problems [3]. UWB technology is an RF-based, non-optoelectronic sensing technology that is effective for communication applications like activity monitoring, tracking, and human localization [4,5]. Attractive advantages of UWB include interoperability with other technologies, compact antenna design, robustness against multipath interference, low cost and power consumption, and high temporal resolution [6,7]. Optoelectronic sensors have been widely used for therapeutic exercises classification, human activity detection, motion capture, and pose estimation [8,9,10]. These sensors face significant challenges, including privacy concerns, susceptibility to occlusion, and sensitivity to environmental light [3]. Commercial inertial measurements units have also been used for activity, motion, and pose detection. But, inertial sensors interfere with drift.

In contrast, UWB radar, a non-contact ambient sensor, can be used as an alternative to overcome the issues in optoelectrical sensors. With the advancement of machine learning algorithms, it is possible now to make correct predictions using sensor data, particularly in classification problems.Evidently, UWB-radar-based systems have been widely developed for medical diagnosis support, particularly for vital sign detection [11] and human activity detection [12]. For the detection of sports activities and physical exercise recognition, UWB radars have been used in both laboratory environments and in on-body placements, respectively [13,14]. In light of this research, it is feasible to extend our work to the recognition of therapeutic exercises. To the best of our knowledge, no systemcurrently exists that recognizes therapeutic exercises using a single UWB radar in real environments with high accuracy

### 1.1. Significance of Research

This research work proposes a noncontact approach to address the challenges of classifying therapeutic exercises effectively using a single UWB radar in a real environment. UWB radar technology is extensively used in many domains, including in medicine for vital sign monitoring and human activity recognition, but its potential for therapeutic exercise recognition is mostly undiscovered. This research addresses a significant gap in the literature by aiming to classify therapeutic workouts in natural settings using a single UWB radar. This has the potential to completely transform rehabilitation procedures by offering a low-cost, non-intrusive monitoring system. The UWB-based system can be used in clinical and domestic environments. Additionally, using UWB radar data, the research attempts to determine the best feature extraction methods and machine learning models by assessing the practicability and effectiveness of the data obtained from therapeutic exercises using a UWB radar.

### 1.2. Major Contributions

UWB Radar is widely used in many fields of life, particularly in the medical field for the detection of vital signs. Its applications can still be explored in human activity detection, human pose estimation, human physical exercises classification, and sports activity recognition. In these studies, experiments were performed in a controlled environment by implementing multiple UWB radars or in combination with optical and non-optical senses. But, the real challenge lies in developing a system that recognizes therapeutic exercises in a real environment using a single UWB radar. In this study, it we attempted to achieve the above-mentioned challenges and contribute the following:

1. This study was conducted to evaluate the feasibility and efficiency of UWB radar data for classifying therapeutic exercises. In this context, data were collected from the upper extremities of 34 healthy volunteers, under the guidelines of a physiotherapist.
2. Signal processing techniques were used to pre-process and improve the raw UWB data. Afterward, feature extraction methods were used to extract the relevant spectral features. Multiple machine learning (ML) models, such as ridge regression, random forest (RF), gradient boosting machines (GBM), AdaBoost, Gaussian Naïve Bayes (GNB), and an ensemble technique, were trained for recognition.
3. The study presents a novel approach for features fusion, which involves mixing temporal and class prediction probability features retrieved from the temporal and spectral features dataset. The integration of these characteristics enhanced the performance of the machine learning models.
4. The effectiveness of the suggested method was evaluated using metrics such as accuracy, precision, recall, and the F1 score. K-fold cross-validation was performed to assess the reliability of the technique.

The subsequent sections of the article are organized in the following manner. Section 2, provides a thorough examination of the relevant literature. Section 3 provides a detailed explanation of the methods used in this work, while Section 4, contains the experimental results. Finally, Section 5 provides the conclusion.

## 2. Literature Review

Camera-based optical systems, such as Vicon and Microsoft Kinect, are considered the gold standard. However, they can be challenging to use due to privacy concerns [3]. In contrast, a non-optical and non-contact ambient sensor that has no such issues is the XeThru UWB radar [4,15]. This UWB radar can be used for the recognition of therapeutic exercises while also preserving the privacy of subjects. For example, [14] extracted on-body channel information using UWB radar and performed classification of physical exercises. UWB radar has been used in many fields, such as X-Ray vision with material-penetrating radars [16], remote medical patient monitoring [15], radar safety systems for vehicles [17], imaging radar for the detection of concealed weapons [18], sense-through-the wall radar systems, and long-range UWB radar imaging [19]. Many researchers have used it to monitor human behavior during specific situations or events and have developed CASAS to analyze these behaviors. Ref. [5] presented a novel sensing technique for non-wearable UWB radar sensor-based human activity detection. The study was performed with a UWB radar placed on the robot mobile and recognized five activities: lying, sitting with the legs on a bed, sitting on a bed with legs on the floor, standing position, and during walking in an apartment (control environment). Principle Component Analysis (PCA) and Latent Discriminate Analysis (LDA) were used for feature extraction from the raw data of the UWB radar. Finally, long short-term memory (LSTM), a deep learning approach, was implemented to classify the above-mentioned five exercises with an accuracy 99.6%. A thorough analysis of UWB radar on-body channel linkages throughout a range of physical activities, with an emphasis on upper and lower limb range-of-motion movements, was presented by [4]. In this study, a UWB radar was used to demonstrate how types of activities, transmitter–receiver distance, body obstacles, and antenna location and orientation affected channel variances. Kurtosis was found to be useful as a differentiating metric with a scoring accuracy of 98% when recognizing activities of daily living using a UWB radar. Recognizing routine activities is a major goal of smart homes. Ref. [20] offered a simplified method to recognize activities using deep learning models, three UWB radars, and a voting mechanism. The experiment was conducted in a LAIRA’S apartment to capture 15 distinct activities and it achieved a classification accuracy of 90%. For the purposes of elderly care, a study was performed by [12], in which a non-intrusive UWB radar approach was used in an apartment that was being developed as a smart home monitoring system. Time domain features of skewness and kurtosis were extracted from the raw data of the UWB radar. It used a k-nearest neighbor machine learning classifier to recognize four human activities: walking from the entrance toward the radar and back; walking from the entrance to a chair, sitting for a while, then standing up and returning to the entrance; and walking to the middle of the room and falling down. This study achieved an accuracy of 99%. Ref. [21] investigated the viability of identifying Activities of Daily Living (ADLs) in smart homes (LAIRA’S environment) using an UWB radar usinfg a contactless approach. A dataset of 15 distinct ADLs, sleeping, drinking, putting on a coat, preparing pasta, cleaning, preparing tea, doing the dishes, brushing teeth, washing hands, reading a book, L-walk, K-Eat, N-Take meditation, M-put on shoes, and O-Use computer was captured.Time domain features such as minimum, maximum, mean, standard deviation, variance, skewness, kurtosis wave length, mean absolute deviation, energy, crossing correlation, mean crossing rate, and frequency domain features Discrete Wavelet Transform (DWT) were prepared from the raw radar data. Finally, three machine learning models—Classification & Regression Tree (CART), K-Nearest Neighbor and Random Forest—were used for classification purposes and the Random Forest classifier was found to be the best with an 80% accuracy, 79% F1-Score, and 77% Kappa.

Human motion is another kinect behavior that is required to be recognized in many real-life AI applications. UWB radar is also used for the assessment of human motion recognition. Ref. [22] conducted a study to recognize two categories of motions, in situ motion and not in situ motion, using a UWB radar in a laboratory environment. Empirical and PCA-based features were retrieved from the collected raw radar data. K-nearest neighbor and Bagged Tree classifiers were used for the purpose of recognizing motions with 94.4% accuracy for the in situ and 95.3% for the not in situ motions. A Multi-Classification algorithm was developed by [23], a UWB radar was implemented to recognize 12 motions: bowing, jumping upward, falling vertically, sitting down, turning sideways, standing still, crawing, jogging, falling forward, jumping forward, walking, and walking with stick. Data sampling was performed in an apartment. Power Spectrum and Doppler shift features were used to recognize the above-mentioned motions. The classification was performed uisng two machine learning classifiers—K-nearest Neighbors (KNN) and Google LeNet—with five-fold cross validation. This study found KNN to be the best classifier with an accuracy of 98%. Ref. [24] used a contactless approach with a UWB radar for recognizing five motions: straightening the upper body, bending over, sitting down and standing up, turning the upper body in one direction, and stretching the arm up and down. The features engineering technique was used to extract the enveloped frameset from the raw radar data, then it added the raw radar data and converted these findings into (red, green, and blue) RGB images. Resnet-18, Resnet-101, and inspection-Resnet-v2 were trained and achieved 99%, 99.20%, and 96.40% accuracy, respectively. Human motion detection using a UWB radar through the wall was conducted by [25]. The purpose of the study was to classify motions such as walking, standing still, and empty spaces. It proposed UWB radar signals could be used in grids and that grid information could be converted into features. Then, a Random Multimodal Deep Learning framework, optimized by the Spotted Grey Wolf Optimizer (SGWO), was implemented to identify the above-mentioned human motions with 95% accuracy, 0.20 mean square error (MSE);], a highest True Negation Rate (TNR) of 0.95, and a highest True Positive Rate (TPR) of 0.95 being recorded.

UWB radar applications can be used for the detection of human poses. Ref. [26] conducted a study to recognize 10 hand gestures by placing two UWB radars—one on the right hand and other on left hand. The experiments were executed in an apartment. Impedance variation was extracted as a feature and was further converted into grayscale spectrogram images. The transfer learning technique was practiced using two pre-trained models—Alex-Net and VGGA-16. Later, it was found that the best classifier had an accuracy 94.6%. Ref. [27] performed through-wall human posture classification using a Stepped frequency continuous wave (SFCW) radar, a form of UWB radar.In this study, three human poses, empty, standing, and sitting, were included and two apartments were used. Raw radar data were converted into Inverse Fast Fourier Transform (IFFT) as the features. Convolutional Neural Networks (CNNs) used for the recognition of human poses and results revealed 98.56% accuracy. Fourteen hand gestures were also classified by [28] using deep learning methods. In this study, variations in hand movements at different distances and movements were used as the features. This study was conducted in an apartment. For the recognition of hand gestures, three deep neural network models, 3D CNN, 2D CNN and LSTM, were trained. LSTM was found to be the best with a 96% accuracy score. A recent study was presented [29], in which the 3D-TransPOSE model was used with UWB radar signals for the prediction of 3D human keypoints.

A UWB radar was used in the body-contact approach used for the recognition of physical exercises by Ref. [14]. They presented an approach that used four UWB radars to capture the data for 11 upper limb exercises. Physical exercises were performed in an apartment. The 8-by-8 MIMO antenna array of the UWB radar system was operated at a low frequency range. For classification purposes, the statistical kurtosis parameter of line of sight (LOS) and nonline of sight (NLOS) was implemented and it archived a precision greater than 98. 0%. UWB radars were used for the recognition of sports activity along with a combination of inertial sensors in a laboratory environment. Four sports activities—break, pull-up, squats, and dips—were included. The data from three UWB radars and IMU were used as the features. The CNN model was used to recognize the sports activities and it finally achieved an accuracy of 97.5%.

From the literature above and the comparative report on UWB radar for detecting kinect behavior (activity, motion, pose, & exercise), it is evident that UWB radars has been extensively employed in assessment of human activities, motions, poses, physical exercises, and sports activities. Consequently, there is potential for its utilization in the recognition of therapeutic exercises. However, the majority of studies involved multiple UWB radars, and experiments were typically conducted in a controlled environment, such as a LAIRA apartment. The challenge lies in using a contactless approach with a single UWB radar to accurately classify the therapeutic exercises in a real environment. This study aims to address this challenge by exploring appropriate solutions. There is also a need to find the best feature engineering approach, such as the signal-to-noise ratio (SNR) inherent in the UWB radar data.In addition, determining the most suitable machine learning classifier for the identification of therapeutic exercises is imperative for the success of this endeavor.

## 3. Materials and Methods

This section includes various essential elements that seek to clarify the process of obtaining, preprocessing, and engineering features from the data. The “Proposed Methodology” Section 3.1 provides an outline of the comprehensive framework that directs our approach towards the analysis of therapeutic exercise data. Subsequently, Section 3.2 in “Data Collection” provides detailed information on the precise methods used to obtain and carefully choose the data for analysis. Section 3.3, titled “Signal Processing and Feature Extraction” explains the methods used to prepare the raw data and extract important features that are essential for further analysis. Section 3.4 introduces a novel methodology that aims to improve the effectiveness of machine learning models by combining temporal features and class prediction probability features collected from the temporal and spectral feature dataset.

### 3.1. Proposed Methodology

**Figure 1:**
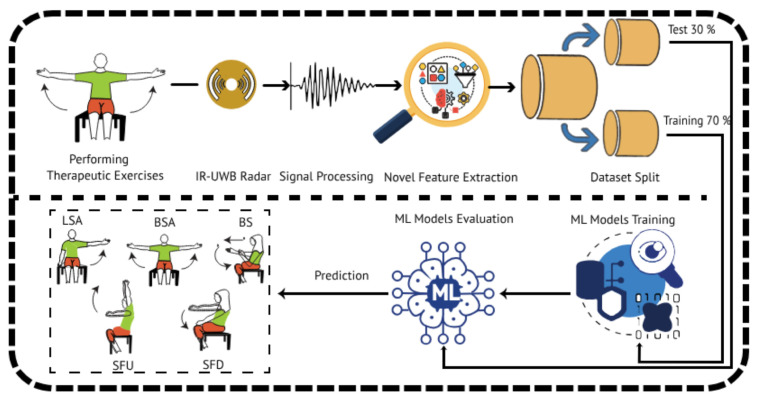
The proposed enhanced research methodology for recognizing therapeutic exercises.

In this section, a novel research methodology is presented to classify the therapeutic exercises. The first phase started by capturing the UWB-radar-based sensor data while the subjects were performing the exercises, as shown in Figure 1. The second step involved applying advanced signal processing techniques to the radar-based dataset to eliminate noise and ensure the integrity of the acquired data. In the third step, an innovative method of feature engineering that integrated temporal features and class prediction probability features was applied. This resulted in a comprehensive feature set that successfully represented the required characteristics of the signal. In the proceeding phase, 70% of the dataset was set aside for training and the remaining 30% was used for testing. By verifying the performance of the model using the remaining 30% of the test data that had not yet been included in the training dataset, the efficacy and generalizability of the machine learning models that were created were evaluated. After rigorous performance testing, the ML model that demonstrated a higher efficacy and accuracy in the recognition of therapeutic exercises using UWB radar data was selected for the classifying task.

### 3.2. Data Collection

This study was carried out with meticulous attention to detail, adhering to ethical principles and receiving approval from the Ethics Committee of the Khawaja Fareed University of Engineering and Information Technology (KFUEIT). The Ethics Committee evaluated all of the mandatory aspects needed in order to proceed with this study and assured that the entire research work was conducted under the principles specified in the Declaration of Helsinki. The primary emphasis was to observe ethical standards by protecting the welfare, rights, and privacy of all participants in the research work. The data collecting process was performed in the ICT building of Khawaja Fareed University of Engineering and Information Technology, Rahim Yar Khan, Punjab, Pakistan, observing and practicing ethical and research standards. The study focused on recognizing five therapeutic exercises using single UWB radar. Overall, 34 participants were included in the study, comprising 34 males, and the age range was between 21 to 29 years old.All of the participants were normal, healthy people. All of the subjects participated voluntarily, and a consent form was filled in by every subject prior to participation.

**Figure 2:**
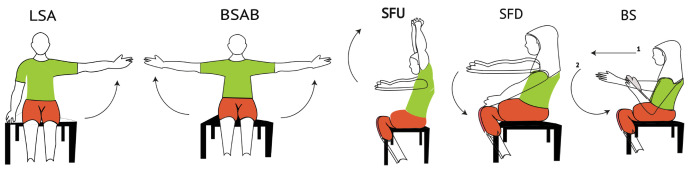
Therapeutic exercises and the method of performing them.

In this study, therapeutic exercises including abduction of the left shoulder (LSA), bilateral abduction of the shoulder (BSA), flexion of the shoulder up (SFU), flexion of the shoulder down (SFD), and breaststroke (BS) were used, as shown in Figure 2.

These exercises are used to treat injuries and illnesses in the upper extremities—more details are presented in Table 1.

**Figure 3:**
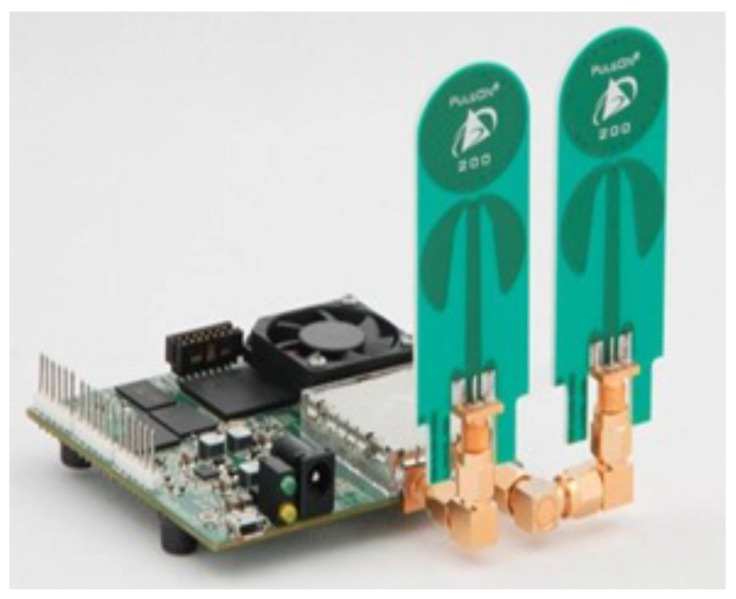
PulseON time domain 410 (p410) UWB radar.

In this study, we used te PulseON time domain 410 (p410) UWB radar, a compact impulse-radio UWB radar system placed on a chip, as depicted in Figure 3.

**Figure 4:**
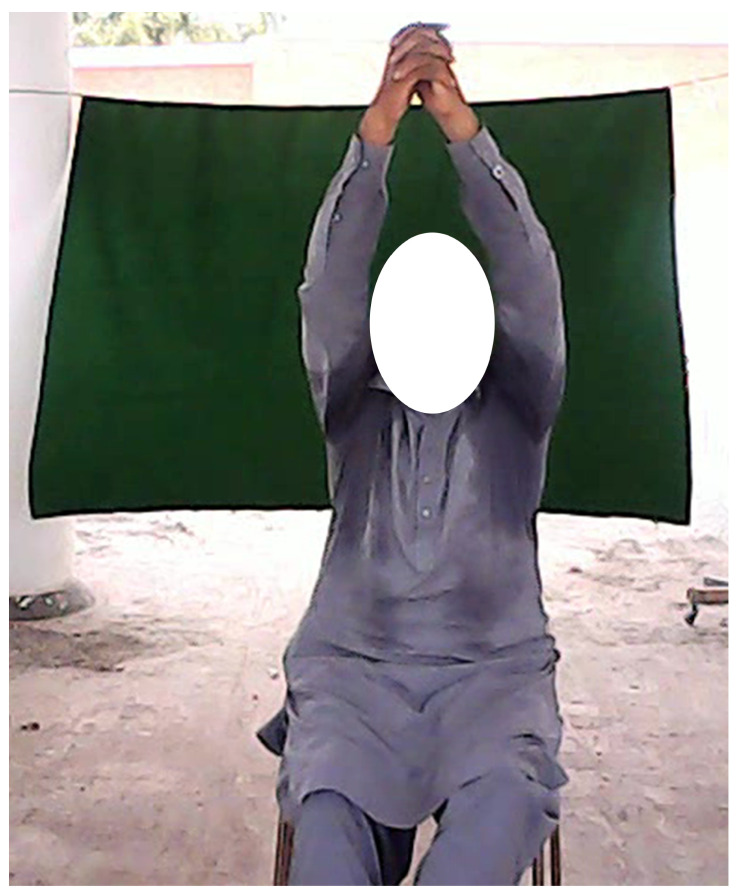
Subject Performing LSA excercise.

The UWB radar system used a monostatic configuration, with separate transmit and receive antennas placed close together. The apparatus complied with the Federal Communications Commission’s (FCC) guidelines for radio wave emissions between 3.1 and 5.3 GHz. With a bandwidth of 2.2 GHz, the waves that were released had a central frequency of 4.3 GHz. To determine the feasibility of using a single UWB radar as the exclusive technology for the recognition of therapeutic exercises in a real environment, a thorough testing protocol was practiced in this study. Upon the recommendation of the physiotherapist’s guidance, 5 therapeutic exercises, LSA, BSA, SFU, SFD and BS, relevant to the upper limbs, were selected as the initial experiment. Details of the therapeutic exercises and their rehabilitation purpose are mentioned in Table 1. The UWB radar was set up at a distance of 2 m and placed on a stand at a height of 94 cm (at the level of subject’s chest). Each participant performed each exercise for 15 s and repeated the same exercise 10 times—this created a comprehensive dataset. During the exercise session in a real environment, as shown in Figure 4, a rest time was allowed in order to reduce fatigue and boost the energy of the subject, as prescribed by the therapeutic medication standard.

### 3.3. Signal Processing and Feature Extraction

The therapeutic exercise data were captured in comma separated value (csv) file format, the 15 s radar scanned sensation was recorded as a matrix (120 rows, 1440 columns), capturing 20 rows/s, where each column represented a vector that indicated the radar return signal in the fast time domain. Similarly, every row of the dataset represented the signal in the slow time domain. The therapeutic exercise movements can be detected by calculating the distance of the subject from the radar, using the following formula:

Total scanned distance in centimeters = 950 cm

Total number of columns = 1440 bins / micro-doppler signature

Distance per column covered = 950/1440 = 0.659 cm

The subjects stood at a distance of 2 m, so the upper limb movement can be detected in between 259 columns to 320 columns (62 columns represent therapeutic exercise movement). The radar scan outputs are formatted as a matrix, where each column corresponds to a UWB radar return signal vector spanning a distance of 0.659 cm from the radar. Given the estimated distance of the subject’s upper limb movements from the radar, approximately 2 m (200 cm), a column range from 259 to 320 was selected to capture the relevant movement patterns. This range was presumed to include the area where signals associated with the therapeutic exercises were most likely to be detected. To mitigate clutter effects, the acquired data were processed using a two-pulse canceller, as described in Equation (1).

(1) $$R_{\mathrm{output}}=R_{i}-R_{i-1}$$

**Figure 5:**
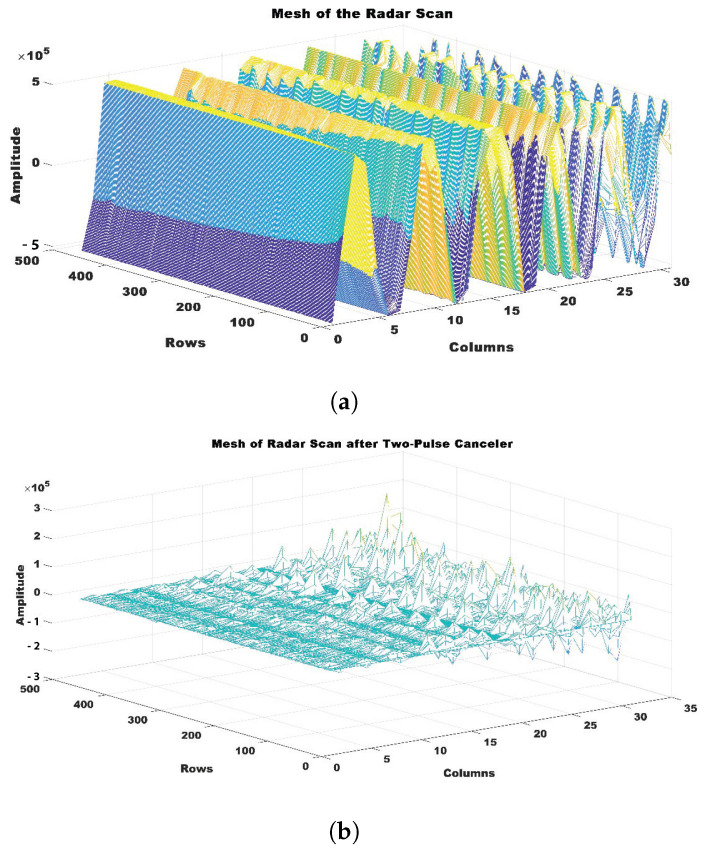
Representation of pulse canceler effects: (**a**) radar scan prior to the implementation of a pulse canceler, and (**b**) radar scan after the application of a pulse canceler.

The resulting value, $R_{\mathrm{output}}$, was the result of subtracting the previous radar return signal $R_{i-1}$ from the present radar return signal $R_{i}$. This technique successfully filtered out noise and other anomalies in the UWB radar data. The improved output then provided a more discernible radar signal, ideal for further processing. The radar scan fragment is shown in Figure 5, both before and after the pulse canceller was applied.

**Figure 6:**
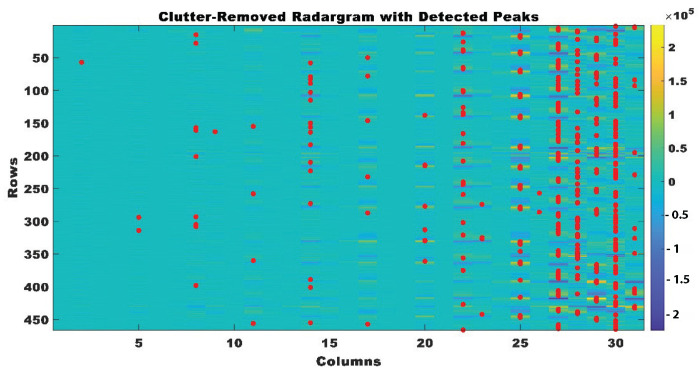
Clutter removed UWB radar-grams with peaks detected.

By first identifying the peaks in the UWB radar data and then filtering out irrelevant information, the physiological windows were defined. Key motions in the therapeutic activities, including body parts approaching or retracting from the radar, were detected using the peak detection algorithm as significant peaks in the radar data. The presence of signals related to therapeutic movements was indicated by values surpassing a threshold of $0.4\times 10^{4}$, which was utilized to differentiate these peaks. Figure 6 shows the peaks identified after the elimination of clutter.

**Figure 7:**
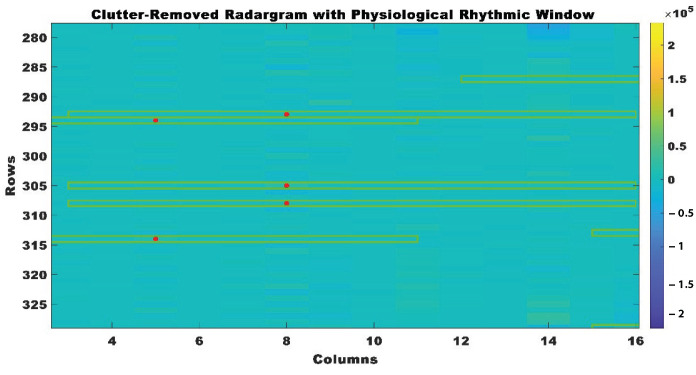
Clutter removed radar-grams with extracted physiological windows.

The physiological window was created by extending values around the observed peaks, resulting in a section of the radar signal that encompasses body movements related to therapeutic exercises, with the recognized peak at its core. A fixed size of 10 was used to compute each window, which consisted of a range of values adjacent to the peak. In order to guarantee the window remained within the radar data bounds, the commencing index was determined by subtracting half of the window size from the peak index. This allowed for the inclusion of data points that preceded the peak without extending beyond the start of the data. In contrast, the peak index was used to determine the ending index, which was then multiplied by half of the window size. By staying inside the radar data range, this method retained the window’s validity and properly piqued the relevant peak values. The physiological window was formed using the created window, which was centered on the peak, as shown in Figure 7. Iteratively applying this procedure to each observed peak generated a sequence of windows that depicted the therapeutic exercise motions. By using this method, it was much easier to analyze the body’s motions, which in turn helped pinpoint the most effective therapeutic activities.

**Figure 8:**
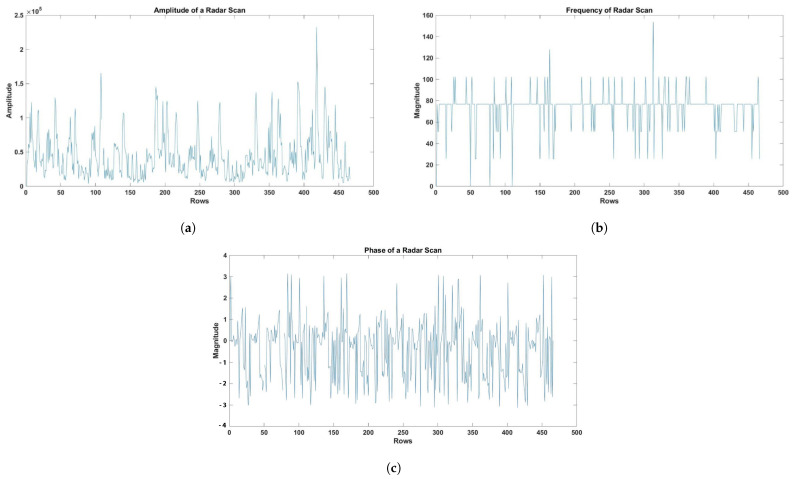
(**a**) Amplitude (**b**) Frequency (**c**) The phase of the radar scan.

The therapeutic exercise signals were characterized and evaluated by deriving distinct properties for each movement of the exercise. In order to find the maximum amplitude of each signal, the highest value within each session was determined. Higher amplitudes indicated more noticeable movements and lower amplitudes indicated more delicate movements; variations in amplitude acted as markers for the presence or absence of therapeutic movements. In order to examine and differentiate the signal’s frequency components, Fast Fourier Transform (FFT) was used to study the frequency characteristics of each therapeutic exercise movement. Further, FFT was used to extract the phase component, which shed light on the therapeutic activities’ temporal components, such as the timing and synchronization of various frequency components in the signal. In order to provide a complete picture of the therapeutic exercise signals obtained from the radar data, the calculated characteristics were averaged over the physiological rhythmic windows. To make sure the results were similar and relevant, the characteristics were normalized to account for differences in signal magnitudes. The approach was used to obtain the amplitude, frequency, and phase components of the radar scan, as shown in Figure 8. The efficiency of amplitude, frequency, and phase in accurately recording human body activity, posture, and movement led to their selection as crucial features [21,27].

The amplitude, frequency, and phase related to each therapeutic exercise rhythmic window were used to extract a set of valuable attributes: amplitude, amplitude mean, amplitude standard deviation (amplitude SD), amplitude range, phase, phase mean, phase standard deviation (phase SD), phase range, spectral centroid frequency (SCF), spectral spread frequency (SSP), spectral skewness frequency (SKNS), spectral kurtosis frequency (SK), spectral entropy frequency (SEF), spectral flatness frequency (SFLT), spectral crest frequency (SCF), spectral flux frequency (SF), spectral slope frequency (SSL), spectral decrease frequency (SD), and spectral rolloff frequency (SRF). The extraction of such aspects from a radar signal was highly significant, as it allowed for gaining valuable insights into the inherent distinctive qualities of the signal. Table 2 provides the specific information on these qualities. In addition, a label was assigned to each extracted feature set to indicate the category of the accompanying radar scan. For example, LSA:1, BSA:2, SFU:3, SFD:4, BS:5. The feature sets, together with their corresponding labels, were then stored in a CSV file.

### 3.4. Proposed Feature Engineering Approach

**Figure 9:**
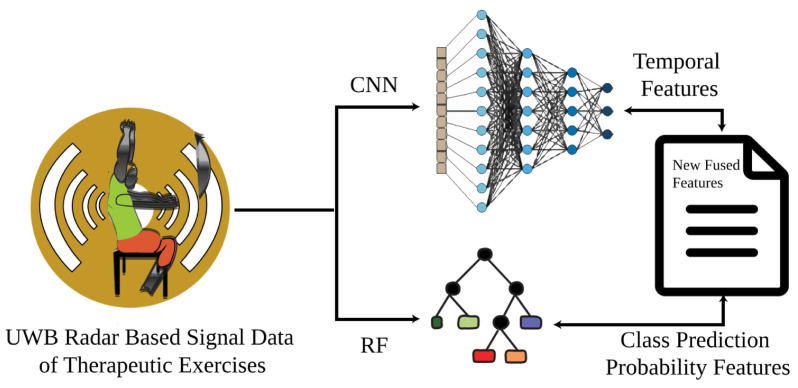
The workflow demonstrates the novel proposed feature fusion technique.

The research presents a novel technique for feature engineering designed to recognize therapeutic exercises using UWB radar signals. This feature extraction approach is an essential component of the proposed system. Figure 9 illustrates the application of this feature engineering technique. The parameters of the CNN and RF models, as mentioned in Table 3, were carefully selected through a series of iterative experiments.

CNN was selected for extracting important temporal features because of its efficacy in capturing local patterns, which are crucial for assessing the dynamic aspects of therapeutic exercise data. Concurrently, the pre-processed dataset was fed into the RF model to extract features associated with the probability of predicting classes. RF, renowned for its ability to effectively handle intricate datasets, offers significant insights regarding the probability of connections within various categories. By leveraging the strengths of both models, the design achieved feature fusion. A novel feature set was created by merging the temporal prediction probabilities from CNN with the class prediction probabilities from RF, as shown in Figure 9. This fusion maximizes the discrimination among classes and minimizes intra-class variance, resulting in outstanding effectiveness for identifying therapeutic exercises. The specific implementation of the feature fusion technique in Figure 9 involves extracting temporal features using CNN, which captures the local patterns and dynamics of the therapeutic exercises. Simultaneously, the dataset is processed by the RF model to generate class prediction probabilities. These probabilities are then combined to form a comprehensive feature set, enhancing the model’s ability to discriminate between different classes and improve the overall recognition accuracy.

### 3.5. Employed Machine Learning Models

In emerging AI technologies, machine learning is widely used in medical sciences and the physiotherapy industry. Machine learning services are employed for diagnosis assistance, treatment planning, patient monitoring systems, and cancer disease prediction. In light of the above-mentioned research studies, machine learning algorithms can be applied for the recognition of therapeutic exercise data acquired from UWB radars. In this study, the Ridge Classifier, RF, Gradient Boosting Machine (GBM), Ada Boost, Naïve Bayes classifiers, and EnsembleRRGraBoost ensemble methods were also trained. The accuracy and efficiency of machine learning models is reliant on hyperparameter tuning. To identify the appropriate parameters, a grid search technique was employed in this study. The most effective performance of each machine learning classifier was evaluated based on the parameter values to recognize therapeutic exercises. The overall objective was to enhance the classifier accuracy, recall, precision, and F1 score. Table 4 shows the machine learning classifiers along with their best explored parameter values. This is a significant detail that impacts the performance of machine learning models.

## 4. Results and Discussion

**Figure 10:**
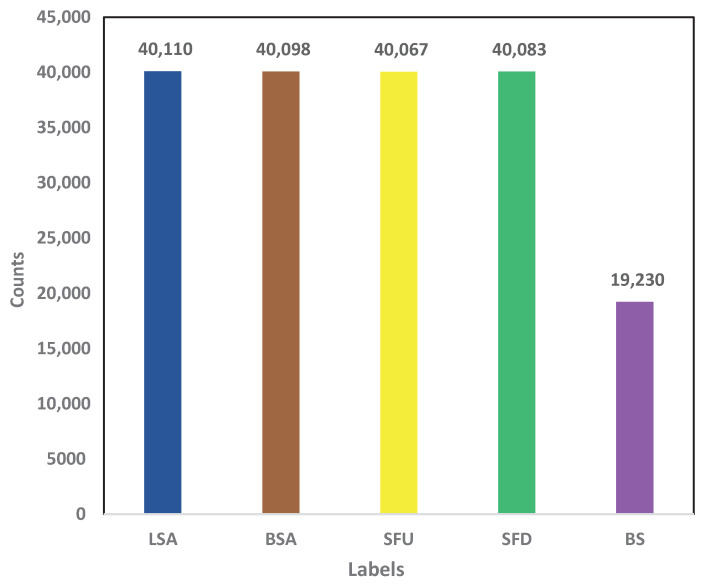
Distribution of class-wise samples.

Machine learning methods are widely used for the recognition of human activities, human motion recognition, pose estimation, and the classification of physical exercises. In this study, machine learning methods were applied for the recognition of therapeutic exercises based on a UWB- radar-based detected dataset. The performance of the machine learning model was measured using essential performance metrics of accuracy, precision, recall, and F1 score. The accuracy metric assessed the general accuracy of the machine-learning-based detection system, whereas the prevision and recall performance metrics dealt with correctly classifying each therapeutic exercise by minimizing the occurrence of false positives and false negatives. The F1 score depicted the balance between precision and recall. The F1 score also provided balanced information about athe machine learning model, demonstrating its effectiveness in cases of imbalanced datasets. The distribution of labels revealed valuable information about the composition of the dataset and the distribution of therapeutic exercise samples. In this study, a relatively equal representative, well-balanced dataset including five therapeutic exercises, LSA, BSA, SFU, SFD, and BS, was collected, as depicted in Figure 10. This helped achieve accurate and reliable outcomes from machine learning models.

### 4.1. Data Splitting

**Figure 11:**
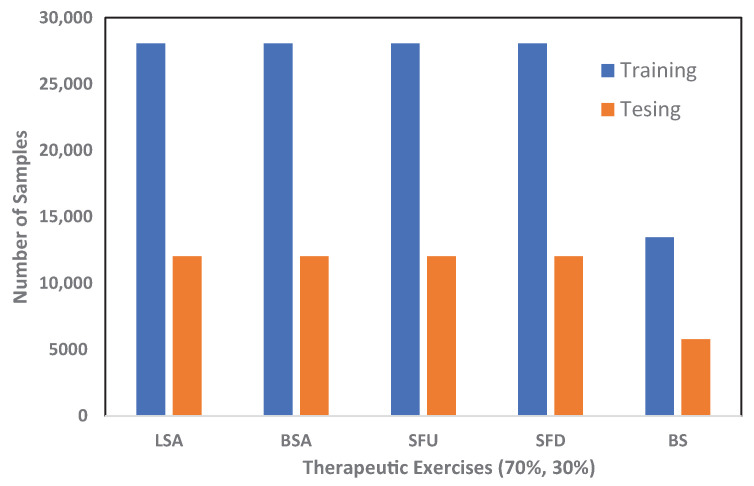
Data splitting for each therapeutic exercise.

**Figure 12:**
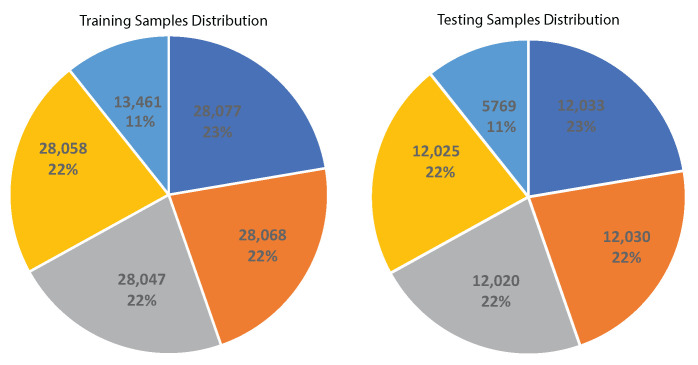
Sample distribution in the training and testing dataset.

The dataset was divided into two sets, 70% and 30%, for training and testing purposes respectively. Data distribution was achieved using the stratify sampling technique to ensure equal representation of each class. The 70% training dataset comprised 12,5711 total samples distributed as follows for each therapeutic exercise: LSA 28,077, BSA 28,068, SFU 28,047, SFD 28,058, and BS 13,461. The 30% test dataset included 53,877 total samples, distributed as follows for each therapeutic exercise: LSA 12,033, BSA 12,030, SFU 12,020, SFD 12,025, and BS 5769, as depicted in Figure 11 and Figure 12. This is a systematic methodology was used to create a well-rounded dataset for therapeutic exercise recognition.

### 4.2. Results Using Temporal and Spectral Features

The features used in the study included amplitude, frequency, and phase-related attributes extracted from the rhythmic window for each therapeutic exercise.This study utilized eight machine learning models to evaluate their categorization capability on a preprocessed dataset derived from UWB radar signals. Table 5 displays a succinct summary of the performance metrics for six models: Ridge; RF; GBM; AdaBoost; GNB; and an ensemble method, EnsembleRRGraBoost; DNN; and CNN.

**Figure 13:**
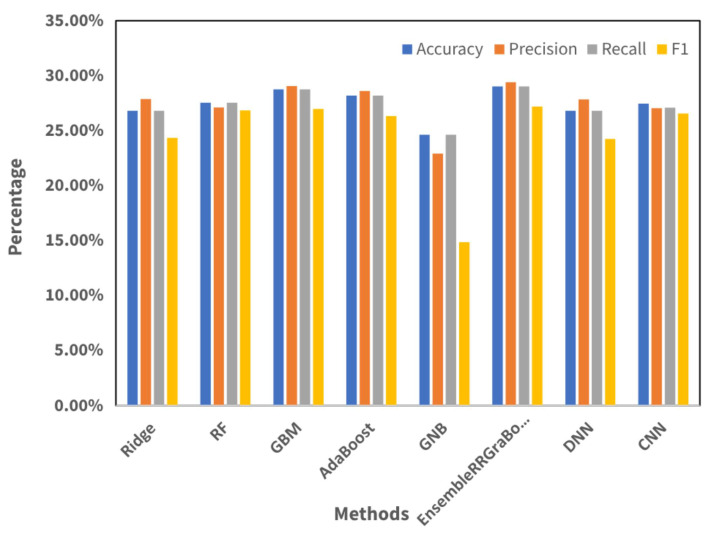
Visualization performance metrics acquired on the original feature set.

GBM outperformed other models, exhibiting a superior accuracy, precision, recall, and F1 score. This demonstrated the efficacy of GBM for accurately classifying events based on the initial set of characteristics. AdaBoost closely followed GBM, demonstrating an excellent performance for all aspects. Although the RF and Ridge models produced similar results, they exhibited a slightly worse performance relative to GBM and AdaBoost. The performance of GNB, however, showed a significant reduction, marked by a reduced precision and F1 score. The ensemble model, which synergistically combined the advantages of Ridge Regression and Gradient Boosting, exceeded all individual models in terms of the projected accuracy, precision, recall, and F1 score. This highlights the advantages of using multiple basic models to enhance the overall precision of forecasts, as depicted in Figure 13. Among the neural network models applied for classification, DNN achieved an accuracy, precision, recall, and F1 score of 26.81%, 27.87%, 26.81%, and 24.35%, respectively, and CNN achieved 27.54%, 27.11%, 27.54%, and 26.86%, respectovely, in the same measures. Despite the comprehensive set of features extracted, the accuracy reported in Table 5 was lower than expected. This lower accuracy indicates that the individual features alone were not sufficient to provide an optimal classification performance. The feature fusion approach was employed to address this limitation by combining multiple features to improve the recognition of therapeutic exercises.

These findings highlight the importance of using feature selection and model combination strategies to optimize classification issues. By utilising ensemble methods and selecting appropriate algorithms, it was possible to increase the precision and robustness of the classification models. Hence, by enhancing their practical applicability, further exploration might involve fine-tuning model parameters, exploring other feature engineering strategies, and assessing the model performance on new datasets based on feature fusion techniques to verify their ability to generalize.

### 4.3. Results with Proposed Feature Engineering

**Figure 14:**
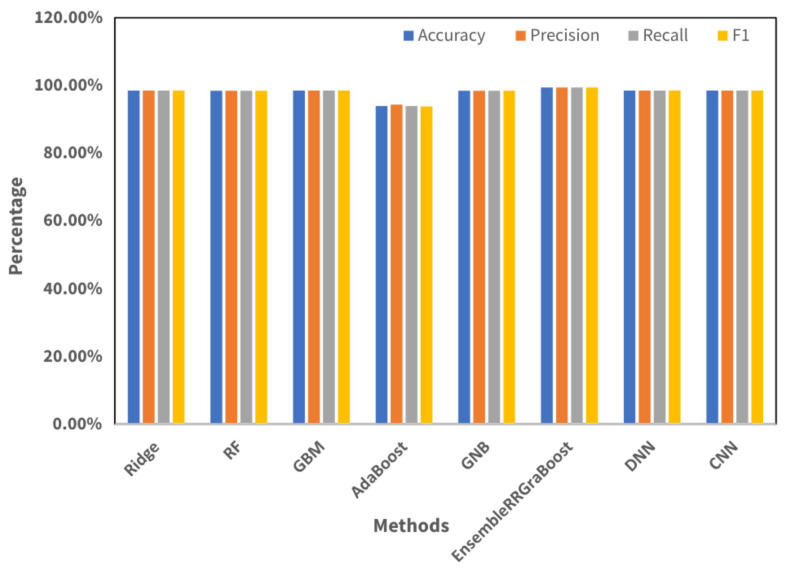
Visualization performance metrics, recorded on fusion features set.

This section presents the results measured through the innovative feature fusion method, based on the proposed feature engineering technique in our study. The utilization of innovative feature engineering techniques has led to substantial improvements in the efficacy of all machine learning models, as depicted in Table 6 and visualized in Figure 14.

This further validates the efficacy of ensemble methodologies for optimizing forecast precision. To summarize, the results unequivocally demonstrated that the proposed feature engineering procedures significantly enhamced the precision and dependability of machine learning models for classification tasks. Further work may involve enhancing the engineering aspects and assessing the model performance on diverse datasets to verify their potential for widespread application in the recognition of therapeutic exercises.

### 4.4. K-Fold Cross-Validations Results

**Figure 15:**
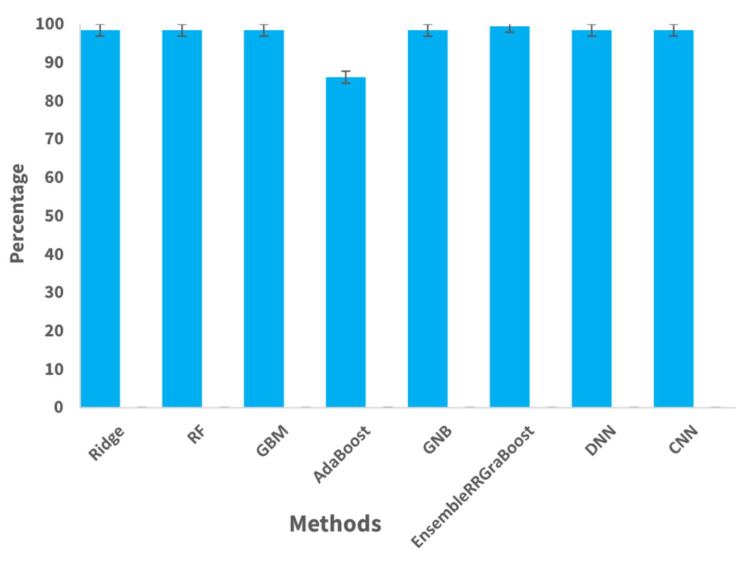
Cross-validation score with standard deviation bar.

The performance evaluation of the recommended machine learning methods was conducted using k-fold cross-validation. The features were divided into five subsets to accomplish validation. The findings shown in Table 7 and their comparison depicted in Figure 15 reveal a consistently high level of precision in the Ridge, RF, and GBM models, with a low variation, suggesting a steady performance throughout many folds of the cross-validation process.

AdaBoost exhibited a somewhat inferior accuracy in comparison with other models, accompanied by a greater standard deviation, indicating a notable fluctuation in performance across various folds. Similarly, GNB demonstrated a moderate level of accuracy with a relatively small standard deviation.

The ensemble model, EnsembleRRGraBoost, consisting of Ridge, RF and GBM, achieved the highest accuracy compared with all of the other models, with a low standard deviation. This suggests that the model performed well consistently over multiple cross-validation folds, suggesting its robustness and stability. The DNN model attained an accuracy of 98.47% with a standard deviation of 0.0012, while the CNN model reached an accuracy of 98.49% with a standard deviation of 0.0013. The findings indicate that both DNN and CNN demonstrated an exceptional performance, with a high level of accuracy and low variation observed across multiple iterations of the cross-validation process. The k-fold cross-validation findings offered vital insights into the generalizability and stability of the machine learning models. The k-fold cross-validation helped to evaluate the models’ performance under different settings and test their effectiveness for classification tasks.

### 4.5. Computation Complexity Analysis

The runtime computation complexity analysis of the applied machine learning models with the proposed feature engineering technique is recorded in seconds, as shown in Table 8. The Ridge Regression algorithm took 2.82 s to execute, mainly due to the computational cost involved in performing matrix operations. The RF algorithm took 2.84 s to complete and involved building multiple decision trees. GBM built trees sequentially to correct errors and completed the work in 2.71 s. The AdaBoost method, which combined many weak learners like decision stumps, had a processing time of 2.19 s, indicating a faster performance. The GNB method had the shortest processing time, accomplishing the challenge in 2.12 s. This could be due to its reliance on clear assumptions on the independence of features. The composite technique, which likely incorporates models such as ridge regression, random forest, and gradient booster machine, took 2.26 s to execute. DNN attained a precision of 98.47% with a standard deviation of 0.0012, while CNN acquired a precision of 98.49% with a standard deviation of 0.0013. The findings indicate that both DNN and CNN exhibited an exceptional performance, demonstrating excellent accuracy and minimum variability throughout multiple folds of the cross-validation technique. The EnsembleRRGraBoost algorithm demonstrated that it took a reasonable amount of time to perform feature engineering in order to precisely classify the instance of therapeutic exercise.

## 5. Discussion

This research investigated machine learning approaches for classifying therapeutic exercises by analyzing a dataset acquired from UWB radar emissions.Because of the inherent complexities in data related to performing therapeutic exercises in a real environment, the initial findings of the machine learning models were limited by the original features. The initial findings of machine learning models were constrained by the original features.The introduction of a novel feature fusion approach that extracted temporal and prediction probability features resulted in a significant advancement. This innovative strategy greatly improved the machine learning model performance.Ridge, RF, GBM, and EnsembleRRGraBoost achieved an accuracy from 26.81%, 27.54%, 28.76%, and 29.02% to 98.48%, 98.43%, 98.47%, and 99.45%, respectively. The utilization of k-fold cross-validation highlighted the robustness and ability to apply the machine learning models to new data. The performance metrics for DNN and CNN showed consistent accuracy across multiple iterations. DNN maintained an accuracy of 98.48% in all four reported instances, while CNN showed a slight variation, ranging from 98.47% to 98.48%. The ensemble model exhibited the highest accuracy and minimal standard deviation, indicating its stability over different folds. The results emphasize the capability of machine learning methods, especially when combined with creative feature engineering, to improve the accuracy and reliability of therapeutic exercise identification systems. This study is limited by its sole focus on upper limb shoulder exercises.Although the therapeutic exercises were performed in a controlled environment, applying these methods in a real clinical setting presents challenges, particularly when multiple subjects are performing the same or different therapeutic exercises simultaneously. Additionally, lower limb therapeutic exercises were not included in this study.

Ridge was the most successful model, achieving superior levels of accuracy, precision, recall, and F1 score compared with all of the other individual models. All of the measures attained a remarkable 98.48%. The RF, Ridge, and GNB models consistently yielded outstanding performance enhancements, reaching an accuracy, precision, recall, and F1 score of over 98%. This demonstrates the effectiveness of the proposed feature engineering procedures in enhancing the predictive capabilities of the models. The performance of AdaBoost exhibited a substantial improvement in comparison with the outcomes obtained using the original features. Although the metrics of this model were still lower than those of the other models, the incorporation of feature engineering led to a substantial enhancement in accuracy, precision, recall, and F1 score. In addition, both the DNN and CNN models demonstrated an outstanding performance, surpassing or obtaining a score of 98.47% for all parameters, including accuracy, precision, recall, and F1 score. DNN attained a 98.48% accuracy across all measurements, while CNN earned a 98.47% accuracy and marginally superior metrics in other domains, showcasing the robustness and durability of these neural network designs. The ensemble model, which combined Ridge Regression and Gradient Boosting, consistently outperformed all individual models, achieving an extraordinary accuracy, precision, recall, and F1 score of 99.45%.

Recent advancements in UWB radar feature extraction include time-frequency analysis methods like STFT and Wavelet Transform; deep learning techniques such as CNNs and RNNs; and statistical methods like higher-order statistics and PSD. While these methods provide valuable insights into radar signals, the technique presented in the paper demonstrates several key strengths. It effectively integrates CNNs and RF models, combining temporal feature extraction with probabilistic class prediction, which enhances both the robustness and accuracy of UWB radar signal analysis. The approach excels in feature fusion by merging CNN-derived temporal prediction probabilities with RF-derived class prediction probabilities, leading to an improved classification performance. This method’s adaptability allows it to be applied to various radar signals and therapeutic exercises, showcasing its versatility. It significantly enhances the performance by maximizing class discrimination and minimizing intra-class variance, surpassing traditional methods. Additionally, the comprehensive analysis provided by integrating multiple data sources and feature extraction techniques offers a deeper understanding and greater utility of radar signals, highlighting the paper’s innovative approach and effectiveness for improving UWB radar performance and exercise recognition.

## 6. Conclusions

In this study, a novel approach was developed for the classification of therapeutic exercises using a single UWB radar sensor in a real environment with state-of-art machine learning models. The proposed approach will benefit physiotherapists by allowing them to monitor patients performing therapeutic exercises at physiotherapy centers. It can also be recommended for use by patients in remote areas as part of a Physiotherapy Computer-Aided System (CAD), while overcomming concerns like privacy, body occlusion while using opto-electronic sensors, and discomfort when using physical contact-based technologies. In this work, a UWB radar was used at a specific distance in a real environment to capture the data while the subject performed the therapeutic exercises with ease and comfort. Due to the inherent complexities, such as noise, when performing therapeutic exercises in a real environment, the initial findings of the machine learning models were limited by the original features. The application of a novel feature fusion approach that extracted temporal and prediction probability features resulted in a significant improvement. This innovative strategy significantly improved the machine learning model performance. Ridge, RF, GBM, DNN, CNN, and EnsembleRRGraBoost achieved accuracies from 26.81%, 27.54%, 28.76%, 26.81%, 27.45%, and 29.02% to 98.48%, 98.43%, 98.47%, 98.48%, 98.47%, and 99.45%, respectively. Finally, the ensemble model, consisting of Ridge, RF, and GBM, achieved the highest accuracy, 99.45%, compared with all of the other models, with a low standard deviation. The utilization of k-fold cross-validation highlighted the robustness and ability to apply the machine learning models to new data. Future research will focus on including a broader and more diverse group of participants, encompassing various ages, genders, and health conditions. This will enhance the generalizability of the findings and ensure that the system’s effectiveness is validated across a wider demographic. Additionally, future work will involve gathering more detailed information about therapeutic exercises beyond simple recognition. This will include counting the frequency of each exercise, assessing the intensity (low, moderate, or severe), measuring the angle of rotation (range of motion), and recording the duration (how long each exercise is performed). Plans are also in place to evaluate the system’s performance in a real physiotherapy center with multiple subjects to better simulate actual conditions. Expanding the system’s functionality to include therapeutic exercises for the lower limbs is another key goal. These advancements will provide a more comprehensive assessment and broaden the system’s applicability in various therapeutic contexts.

## Figures and Tables

**Table 1:** Details of the therapeutic exercises included in this study along with their rehabilitation purpose.

Physical Therapy Exercises	Explanation (How to Perform)	Advised for Illness or Injury
LSA	Lifting the left arm laterally from the human body	Rotator cuff injuries, frozen shoulder (Adhesive Capsulitis), shoulder impingement syndrome
BSA	Lifting both arms in parallel, laterally from the human body	
SFU	Raising the arm forward and upward	
SFD	Raising the arm forward and downward	
BS	Simulating circular arm movements akin to swimming	Shoulder rehabilitation, improving range of motion

**Table 2:** Time and frequency domain features and their description.

Feature	Explanation
Amplitude	Amplitude is the quantification of the highest magnitude or intensity of a wave or signal, indicating the distance from its baseline to its peak. The phrase “intensity or magnitude” refers to the level or strength of the oscillation.
Amplitude Mean	Amplitude mean refers to the average magnitude of oscillations in a wave. The signal’s strength is represented by the central value, which is crucial for studying waveforms in different domains.
Amplitude SD	Amplitude standard deviation measures the extent to which individual amplitude values in a waveform deviate from the average amplitude, reflecting the degree of dispersion or distribution in the data.
Amplitude Range	Amplitude range refers to the difference between the highest and lowest values of amplitude in a signal, which indicates the degree of variation in signal strength.
Phase	Phase is a measure of the position of a waveform inside a cycle at a specific moment in time. It is usually expressed in degrees or radians and indicates the relative alignment of the waveform.
Phase Mean	Phase mean is the arithmetic average of the phase values in a dataset. It represents the center tendency of the phase distribution and is important for understanding cyclic events and signal processing applications.
Phase SD	Phase standard deviation is a statistical measure that calculates the extent to which phase values in a dataset deviate or spread out from their average.
Phase Range	Phase range, akin to phase standard deviation, is a statistical metric employed to quantify the variability of phase values in a dataset.
SCF	Spectral centroid frequency refers to the average frequency of a signal. Determining the centroid of a spectrum is crucial in audio processing, voice recognition, and music analysis to characterize the timbre and extract features.
SSP	Spectral spread frequency quantifies the extent of frequency dispersion in the spectrum of a signal, providing information on the distribution and concentration of frequencies.
SKNS	Spectral skewness refers to the measure of the asymmetry of a spectral distribution. It quantifies the degree to which the spectral energy is concentrated towards the higher or lower frequencies.
SK	Spectral kurtosis Frequency quantifies the degree of sharpness or uniformity of the frequency distribution in the spectrum of a signal, offering valuable information about the existence of sudden or abrupt elements.
SEF	Spectral entropy refers to the measure of the randomness or complexity of a signal’s frequency content.
SFLT	Spectral flatness refers to the measure of how evenly distributed the energy is across different frequencies in a signal. Frequency is a measure of the tonality or timbral richness of a signal’s frequency spectrum.
SCF	Spectral crest frequency is a measure of the prominence of the greatest peak in the frequency spectrum. It is calculated by dividing the maximum amplitude in the spectrum by the average amplitude.
SF	Spectral flux frequency measures the change in spectral content between consecutive frames, reflecting variations in energy distribution over time in a frequency spectrum.
SSL	Spectral slope frequency refers to the rate at which the spectral amplitude distribution changes with respect to frequency. It indicates the speed at which energy levels decline or increase across the spectrum.
SD	Spectral decrease refers to the reduction in the intensity or amplitude of frequencies in a sound signal as the frequency increases. Frequency is a measure of how quickly the amount of energy in a spectrum declines as the frequency increases.
SRF	Spectral rolloff frequency denotes the threshold below which a specific proportion of the overall spectral energy is concentrated, signifying the boundary where the high-frequency components of the signal spectrum are attenuated.

**Table 3:** Details of the novel feature extraction approach.

CNN		
**Layer Type**	**Parameters**	**Activation**
Conv1D	Filters: 64, Kernel Size: 3	ReLU
MaxPooling1D	Pool Size: 2	-
Flatten	-	-
Dense	Units: 64	ReLU
Dense	Units: 16	Softmax
**RF**		
n_estimators = 100, Max_depth = 3, random_state = 123		

**Table 4:** Details of the appropriate values for the classifier’s parameters with hyperparameters turning using the grid search technique.

Methods		Parameters with Appropriate Values (Hyperparameter Tuning Using the Grid Search Approach)
Ridge		alpha = 1.0, random_state = 123
RF		Max_depth = 3, n_estimators = 100, random_state = 123
GBM		learning_rate = 0.1, max_depth = 3, n_estimators = 100, random_state = 123
Ada Boost		algorithm = ‘SAMME.R’, learning_rate = 1.0, n_estimators = 50, random_state = 123
GNB		priors = None, var_smoothing = 1e-09
EnsembleRRGraBoost		Base models: RF and GBM.
		RF hyperparameters: Max_depth = 3, n_estimators = 100, random_state = 123
		GBM hyperparameters: learning_rate = 0.1, max_depth = 3, n_estimators = 100, random_state = 123
		Meta Model: Ridge Regression.
		Ridge Regression hyperparameters: alpha = 1.0, random_state = 123
**DNN**		
**Layer Type**	**Parameters**	**Activation**
Dense	64 units	relu
Dense	64 units	relu
Dense	5 units	softmax
**CNN**		
**Layer Type**	**Parameters**	**Activation**
Conv2D	Filters 32, kernel size (3,1)	relu
MaxPooling	Kernel size (2,1)	-
Dropout	0.25	-
Conv2D	Filters 64, kernel size (3,1)	relu
MaxPooling	Kernel size (2,1)	-
Dropout	0.25	-
Flatten	-	-
Dense	128 units	relu
Dropout	0.25	-

**Table 5:** Performance metrics for different methods on Temporal and Spectral Features.

Method	Accuracy	Precision	Recall	F1
Ridge	26.81%	27.87%	26.81%	24.35%
RF	27.54%	27.11%	27.54%	26.86%
GBM	28.76%	29.06%	28.76%	26.97%
AdaBoost	28.21%	28.62%	28.21%	26.34%
GNB	24.64%	22.92%	24.64%	14.86%
EnsembleRRGraBoost	29.02%	29.42%	29.02%	27.20%
DNN	26.81%	27.85%	26.80%	24.25%
CNN	27.45%	27.05%	27.10%	26.56%

**Table 6:** Performance metrics for different methods.

Method	Accuracy	Precision	Recall	F1
Ridge	98.48%	98.48%	98.48%	98.48%
RF	98.43%	98.43%	98.43%	98.43%
GBM	98.47%	98.47%	98.47%	98.47%
AdaBoost	93.92%	94.37%	93.92%	93.78%
GNB	98.45%	98.45%	98.45%	98.45%
EnsembleRRGraBoost	99.45%	99.45%	99.45%	99.45%
DNN	98.48%	98.49%	98.48%	98.48%
CNN	98.47%	98.48%	98.48%	98.48%

**Table 7:** Accuracy and standard deviation for different methods.

Method	Accuracy	Standard Deviation (+/−)
Ridge	98.49%	0.0019
RF	98.47%	0.0012
GBM	98.47%	0.0013
AdaBoost	86.21%	0.0504
GNB	98.47%	0.0018
EnsembleRRGraBoost	99.50%	0.0012
DNN	98.47%	0.0012
CNN	98.49%	0.0013

**Table 8:** The runtime computation complexity analysis of the proposed machine learning methods.

Method	Runtime Computations (Seconds)
Ridge	2.82
RF	2.84
GBM	2.71
AdaBoost	2.19
GNB	2.12
EnsembleRRGraBoost	2.26
DNN	2.62
CNN	2.73

## Data Availability

Data will be provided upon reasonable request.

## Abbreviations

The following abbreviations are used in this manuscript:

AI	Artificial Intelligence
ML	Machine Learning
KFUEIT	Khawaja Fareed University of Engineering and Information Technology
GBM	Gradient Boosting Machine
GNB	Gaussian Naïve Bayes
BSA	Bilateral Shoulder Abduction
SCF	Spectral spread frequency
UWB	Ultrawide band
LSA	Left Shoulder Abduction
SSP	Spectral spread frequency
SFU	Shoulder Flexion Up
SKNS	Spectral skewness
BS	Breaststroke
SK	Spectral kurtosis Frequency
SEF	Spectral crest frequency
SFD	Shoulder Flexion Down
SF	Spectral flux frequency
CNN	Convolutional Neural Network
SSL	Spectral slope frequency
RF	Random Forest
SRF	Spectral rolloff frequency
